# Early Mild Stress along with Lipid Improves the Stress Responsiveness of Oscar (*Astronotus ocellatus*)

**DOI:** 10.1155/2022/8991678

**Published:** 2022-05-09

**Authors:** Noah Esmaeili, Hossein Hosseini, Mahyar Zare, Sobhan R. Akhavan, Artur Rombenso

**Affiliations:** ^1^Institute for Marine and Antarctic Studies, University of Tasmania, Hobart, TAS, Australia; ^2^Department of Microbiology, Pathobiology & Basic Sciences, Faculty of Veterinary Medicine, Razi University, Kermanshah, Iran; ^3^Institute of Aquaculture and Protection of Waters, South Bohemian Research Center of Aquaculture and Biodiversity of Hydrocenoses, Faculty of Fisheries and Protection of Waters, South Bohemian Research Center of Aquaculture and Biodiversity of Hydrocenoses, University of South Bohemia in České Budějovice, Na Sádkách 1780, 370 05 České Budějovice, Czech Republic; ^4^Nelson Marlborough Institute of Technology, H-Block, 322 Hardy Street, Private Bag 19, Nelson 7042, New Zealand; ^5^CSIRO, Agriculture and Food, Livestock & Aquaculture Program, Bribie Island Research Centre, Bribie Island, QLD, Australia

## Abstract

Early-life exposure to mild stressors can assist animals in coping with more stressful events in later life. This study was aimed at investigating how early stress and dietary lipid contents affect growth, hematology, blood biochemistry, immunological responses, antioxidant system, liver enzymes, and stress responses of oscar (*Astronotus ocellatus*) (6.8 ± 0.7 g). Six experimental treatments were HL0Stress (high-lipid diet and without stress), HL2Stresses (high-lipid diet and two-week stress), HL4Stresses (high-lipid diet and four-week stress), LL0Stress (low-lipid diet and without stress), LL2Stresses (low-lipid diet and two-week stress), and LL4Stresses (low-lipid diet and four-week stress). During the ten-week trial, fish fed high-lipid diets grew faster (46.41 ± 4.67 vs. 38.81 ± 2.81) and had a lower feed conversion ratio (2.21 vs. 2.60) than those fed low-lipid diets (*P* < 0.05). After acute confinement stress (AC stress), high-lipid groups had higher survival than low-lipid treatments (81.25% vs 72.92%) (*P* < 0.05). Fish subjected to two-time stress (2Stresses) had a higher survival rate after AC stress (90.63% vs. 62.50%), hematocrit, white blood cell, blood performance, total protein, high-density lipoproteins, cholesterol, triglyceride, alternative complement activity (ACH50), superoxide dismutase, glutathione peroxidase, and alkaline phosphatase levels than those not stressed (*P* < 0.05). Contrariwise, glucose, cortisol, alanine aminotransferase, and aspartate aminotransferase levels were significantly lower in the 2Stresses groups compared with 0Stress fish (*P* < 0.05). Collectively, these findings suggest stressing the signs of adaptation in 2Stresses fish. However, a higher number of early stress events (4Stresses) appears to exceed the threshold of manageable stress levels for this species. In conclusion, the HL2Stresses group outperformed the other treatments in terms of growth, health status, and stress responsiveness. Although fish welfare must be considered, these results suggest that early mild stress can result in a greater survival rate after fish are exposed to later acute stress.

## 1. Introduction

The ornamental fish market is becoming one of the most profitable sections in the aquaculture industry [1]. However, little scientific research has been devoted to the nutritional requirements [2] and understanding the link between stress and welfare in ornamental fish, most likely due to numerous fish species (between 600 and 700 fish species based on FAO 2020 [3]).

Oscar (*Astronotus ocellatus*) is one of the most popular ornamental fish species. Oscar is a carnivorous species, and the crude protein concentration and total lipid in commercially available pellets are 450-500 g/kg and 80-150 g/kg, respectively. However, only a few nutritional studies have been conducted, and they formulated diets with 50% crude protein and 14% total lipid [4]. Lipids play numerous roles in fish metabolism, including energy storage, cell signalling, structural components of cell membranes, fat-soluble vitamins, ATP production, and steroid hormone production [5]. In addition, the composition and quantity of lipids in ornamental fish nutrition play a vital role in improving the number and quality of eggs and larvae, as well as improving reproductive performances and colourations [6]. The adverse impacts of “not-optimum” quantity and quality of dietary lipids in fish nutrition were broadly investigated [5, 7–9].

Early life stress is an interesting phenomenon in biology, believing that early stress experiences can shape behaviour, physiological stress responses, immune responses, and fitness (later in life) of an organism's immediate and future coping style [10]. Although it is well-known that either low or high levels of early stress produce negative impacts (for example, poor immune system responses), the mild stress of the right sort and at the right time brings positive outcomes (low-stress responsiveness) [10]. A few studies in fish focused on this phenomenon in the egg and larval stages. For example, the existence of some kind of short-term memory in innate immune response pathways and “trained immunity” in zebrafish (*Danio rerio*) was observed [11]. Auperin and Geslin [12] reported that applying gentle stress at eyed, hatching, and yolk resorption stages reduce the later cortisol responsiveness of 5-month-old rainbow trout (*Oncorhynchus mykiss*). In addition, repeated short exposure of Atlantic salmon (*Salmo salar*) to stress during embryonic or/and posthatch stages positively changed methylome and transcriptome profiles and eventually increased the growth performance of adult fish when compared to controls [13].

To the best of our knowledge, no studies have been carried out with aquatic species in early life stress to determine the interaction of early stress with nutrition. Three questions were raised to investigate further the hypothesis that early mild stress events are beneficial for fish to cope with future stress challenges. First, the impact of early stress on the final survival rate and physiological responses of fish after acute confinement stress (AC stress) was examined. Next, a scheduled stress time window (every other week) was designed to demonstrate the potential adverse effects of “too much” stress in fish. Finally, the effect of lipid levels on this process was determined by formulating high- and low-lipid diets. Therefore, this study is aimed at investigating the effects of dietary lipid composition and repeated mild early stress, according to the number of stress repeats and their interactions (diet lipid level∗ number of stress) on growth performance, hematology, blood biochemistry, immune responses, antioxidant activities, stress responses, and liver enzymes of juvenile oscar.

## 2. Materials and Methods

### 2.1. Ethics Statement

All procedures involving animals were conducted according to the Tarbiat Modares University protocols, which seek to optimise handling and minimise animal stress [14–17].

### 2.2. Diet Preparation and Experimental Design

Two isonitrogenous (400 g crude protein/kg feed) diets with two different lipid levels (180 and 100 g total lipid/kg feed) were formulated using the Lindo software (Table 1). Ingredients were obtained from a nearby Animal & Aquatic Feed store (Arak, Markazi, Iran). During the ten-week experimental period, three scheduled stress windows (moving net around the tanks for 5 minutes after water exchange on Monday and Friday of proposed weeks) were designed to create without stress, two-time stress, and four-time stress treatments. Table 1 shows the chemical composition of the experimental diets. The six treatments were HL0Stress (high-lipid diet and without stress), HL2Stresses (high-lipid diet and two-time stress at week 2 and week 8), HL4Stresses (high-lipid diet and four-time stress at weeks 2, 4, 6, and 8), LL0Stress (low-lipid diet and without stress), LL2Stresses (low-lipid diet and two-time stress at week 2 and week 8), and LL4Stresses (low-lipid diet and four-time stress at weeks 2, 4, 6, and 8) (Table 2). The dietary ingredients were first dried and were mixed thoroughly to ensure homogeneity of the ingredients. Then, liquid ingredients such as Kilka fish oil and soybean oil were carefully weighed and gradually added. The resulting mixture was ground into 1 mm pellets using a meat grinder (Electrokar EC-1, Tehran, Iran). The pelleted granules were then spread out on a tray and dried in an oven at 60°C for 24-48 hours to 90% dry matter. After drying, the pellets were packed in appropriate packages and stored at 4°C [18].

### 2.3. Fish and Experimental Conditions

A total of 360 oscars (initial weight: 6.8 ± 0.7 g) were obtained from the Abzian Center (Mahallat, Markazi, Iran). Eighteen glass tanks were used in a semirecirculating system with a volume of 100 litres of water, three replicates, and 20 fish per tank. Fish were fed a commercial diet (500 and 150 g/kg crude protein and total lipid, respectively) for 14 days of acclimatisation. During a 10-week experimental period, fish were fed experimental diets to apparent satiation three times a day at 09:00, 14:00, and 19:00 hours. Throughout the trial, 20-30% of the tank water was syphoned out daily to remove faeces and debris. Water quality parameters were monitored regularly and kept at standard levels. The photoperiod was set to 12D : 12L, and the temperature (25 ± 1.0°C), dissolved oxygen (6.6 ± 0.7 mg/L), pH (7.5 ± 0.8), conductivity (280 ± 40 *μ*S/cm), and total dissolved solids (200 ± 10 mg/L) were all measured using a Multi Water Quality Meter (Lutron WA-2017SD, Pennsylvania, USA). ASTM International D1426-08 and D3867-09 were used to measure ammonia (0.49 ± 0.1 mg/L), nitrite (0.029 ± 0.004 mg/L), and nitrate (23.0 ± 2.2 mg/L); all of them were within a standard range.

### 2.4. Growth Performance

After fasting for 24 hours, all fish were anaesthetised by 50-70 ppm clove oil extract at the end of week 9 [7]. Weight gain (WG), specific growth rate (SGR), feed conversion ratio (FCR), daily feed intake (DFI), hepatosomatic index (HSI), viscerosomatic index (VSI), and condition factor (CF) were calculated using standard methods and relationships [19]. In addition, four fish from each replicate were randomly chosen and the blood sample was collected (details are described below). It is followed by liver sampling and weighing. The footnote of Table 3 contains a list of all the formulas used.

### 2.5. Blood Collection and Sample Preparation

Hematology, immune responses, blood biochemistry, antioxidant analyses, and serum enzymes were analysed on four random fish from each tank. To reduce stress, the fish were anaesthetised, as mentioned in the last section, and blood samples were collected quickly by caudal vein puncture using a sterile 1 mL syringe. Blood samples were refrigerated for 2 hours, and the serum samples were separated by centrifuging at 3000 × g at 4°C [7].

#### 2.5.1. Hematology Profile

After diluting the whole blood with Natt, M. P., and C. A. Herrick solution (1 : 200), containing 0.1 g of brilliant cresyl blue, 3.8 g of sodium citrate, and 0.2 mL of 37% formaldehyde in 100 mL of distilled water, RBCs were counted in a Neubauer hemocytometer. Five central compartments in the Neubauer chamber's middle square were counted and multiplied by the total of 10,000. The Neubauer chamber's four marginal squares were used to count white blood cells (WBCs) [20]. The cyanmethemoglobin method was used to determine hemoglobin (Hb). Uncoagulated blood (20 *μ*L) was mixed with 5 mL of Drabkin's solution and left in the dark for 5 minutes. It was then measured (in g/dL) with a spectrophotometer at 540 nm. The microhematocrit method was used to calculate hematocrit (Ht). More than two-thirds of hematocrit capillary tubes were filled with uncoagulated blood. The tubes were centrifuged at 13,000 × g for 5 minutes in a microhematocrit apparatus before Ht values were determined using a specific graded sheet [21]. Mean corpuscular volume (MCV), mean corpuscular hemoglobin (MCH), mean corpuscular hemoglobin concentration (MCHC) [22], and blood performance (BP) [23] were calculated according to the below formula:

(1) $$\begin{array}{c} \text{Mean corpuscular volume }\left(\text{MCV}\right)\;\left(\text{fl}\right)=\left(\frac{\text{Hematocrit}}{\text{RBC }10^{6}/{\text{mm}}^{3}}\right)\times 10,\\ \text{Mean corpuscular hemoglobin }\left(\text{MCH}\right)\;\left(\text{pg}\right)=\frac{\text{Hemoglobin}}{\text{RBC }10^{6}/{\text{mm}}^{3}}\times 10,\\ \text{MCHC}=\frac{\text{Hemoglobin}}{\text{Hematocrit }}\times 100,\\ \text{Blood performance }\left(\text{BP}\right)=\text{Ln Hb}\left(\frac{\text{g}}{\text{dL}}\right)+\text{Ln Ht }\left(\%\right)+\text{Ln RBC}\left(\frac{*10^{5}}{{\text{mm}}^{3}}\right)+\text{Ln WBC}\left(\frac{*10^{5}}{{\text{mm}}^{3}}\right)+\text{Ln TP}\left(\frac{\text{g}}{\text{dL}}\right). \end{array}$$

#### 2.5.2. Blood Biochemistry, Antioxidant Enzyme Activities, Serum Enzymes, and Cortisol

Glucose, total protein, albumin, globulin, high-density lipoproteins (HDL), low-density lipoproteins (LDL), cholesterol, triglycerides, lactate, alkaline phosphatase (ALP), lactate dehydrogenase (LDH), aspartate transaminase (AST), and alanine aminotransferase (ALT) were measured using the kits from the Pars Azmun Company (Pars Azmun, Karaj, Iran). The antioxidant enzymes, including superoxide dismutase (SOD), catalase (CAT), glutathione peroxidase (GPx), and malondialdehyde (MDA), were determined using ELISA kits analysis (ZellBio, GmbH, Germany) [24]. Cortisol levels in serum were determined using commercial kits (ichroma, South Korea) [24].

### 2.6. Nonspecific Immune Parameters

#### 2.6.1. Lysozyme Activity

Gram-positive bacteria sensitive to the lysozyme enzyme method was used to determine serum lysozyme (*Micrococcus lysodeikticus*) [25]. In summary, the egg white lysozyme enzyme was used as a standard, with concentrations of 0, 5, 10, 25, 50, and 100 *μ*g/mL dissolved in phosphate buffer (0.1 M and pH = 5.8). Then, 25 *μ*L of the standard sample and serum from each treatment were poured separately into each 96-well microplate, which had three replicates. Following that, 175 *μ*L of *M. lysodeikticus* suspension in the same buffer (75 *μ*g/mL) was added to each well, immediately mixed with serum samples, and allowed to react at 20°C. The light absorbance of the samples was measured using a microplate reader at 450 nm every 30 seconds for up to five minutes (Awareness Technology Stat Fax 3200, Ramsey, USA) [26].

#### 2.6.2. Alternative Complement Pathway Hemolytic Activity (ACH50)

The hemolysis of rabbit RBCs (RaABC) was used to determine ACH50 [27]. Briefly, the RBCs were washed three times with an ethylene glycol tetra-acetic acid-magnesium-vernal gelatine buffer, with the number of cells adjusted to approximately 2 × 10^8^ per mL of the buffer using Neubarb's lamella. Initially, the 100% lysis value was determined by exposing 100 *μ*L of the aforementioned RaRBC stock to 3.4 mL of distilled water. After that, the serum was diluted (×100) and adjusted to the different volumes ranging from 100 to 250 *μ*L (total volume was adjusted to 250 *μ*L with the buffer). Then, it was allowed to react in small test tubes with 100 *μ*L of RaRBC. The mixture was then placed in a 20°C oven for 90 minutes. For each tube, 3.15 mL of sodium chloride solution was added. Samples were centrifuged at 1600 × g for 10 minutes at 4°C, and then, the optical absorbance of the supernatant at 414 nm was measured by a spectrophotometer (ARUN Technology, UK). The volume yielding 50% hemolysis was used for determining the complement activity of the sample as follows:

(2) $$\begin{array}{c} \text{ACH}50\text{ value }\left(\frac{\text{units}}{\text{mL}}\right)=\frac{\;1}{K\times \left(\text{reciprocal of the serum dilution}\right)\times 0.5}. \end{array}$$

In the above equation, *K* is the volume of serum in milliliters, which causes 50% hemolysis; 0.5 is constant; and finally, the dilution factor in this test is 0.01.

#### 2.6.3. Serum Levels of Immunoglobulin, Complement C3 (C3), and Complement C4 (C4)

Serum immunoglobulin levels were measured by ELISA method using a kit (CUSABIO-CSB-E12045Fh, China) at a wavelength of 450 nm following kit instruction [24, 28]. Serum C3 levels were measured by the ELISA method using a kit (CUSABIO, CSB-E09727s, China) at a wavelength of 450 nm [24, 28]. This assay employs the competitive inhibition enzyme immunoassay technique. The microtiter plate provided in this kit was precoated with a goat-anti-rabbit antibody. Standards or samples were added to the appropriate microtiter plate wells with an antibody specific for C3 and Horseradish Peroxidase- (HRP-) conjugated C3 [24, 28]. The competitive inhibition reaction was launched between HRP labelled C3 and unlabeled C3 with the antibody. A substrate solution was added to the wells, and the colour developed opposite to the amount of C3 in the sample [24, 28]. The colour development was stopped, and the intensity of the colour was measured. Serum C4 levels were measured by fish C4 ELISA Kit (MyBioSource, USA) at a wavelength of 450 nm [24, 28]. This assay employs the quantitative sandwich enzyme immunoassay technique. Antibody specific for C4 was precoated onto a microplate. Standards and samples were pipetted into the wells, and the immobilised antibody was bound to any C4 present [24, 28]. After removing any unbound substances, a biotin-conjugated antibody specific for C4 was added to the wells. After washing, avidin-conjugated horseradish peroxidase (HRP) was added to the wells. Following removal of any unbound avidin-enzyme reagent, a substrate solution was added to the wells and colour developed in proportion to the amount of C4 bound in the initial step [24, 28]. The colour development was stopped, and the intensity of the colour was measured at a wavelength of 450 nm [24, 28].

### 2.7. Acute Confinement Stress (AC Stress) and Mild Stress

AC stress, according to our previous research [29], with some modifications, was used at the end of the experiment to determine the fish ability to tolerate stress. The confinement stress test included twenty-four random fish per treatment (12 fish per tank, two tanks per treatment). Fish were netted and held out of the water for 30 seconds before transferring them to a plastic mesh bucket in their original tank at a density of 120 g/L for 5 hours. Aeration was provided for the fish to prevent oxygen depletion and early death. Blood sampling and serum extraction were performed as previously described at the end of 5 hours of stress (four fish per tank). Table 3 shows the survival rate of fish after 48 hours in various treatments.

Fish were stressed on Monday and Friday in the scheduled weeks (Table 2), moving the net around the tanks for 5 minutes after exchanging water to expose the fish to mild stress.

### 2.8. Statistical Analysis

**Figure 1:**
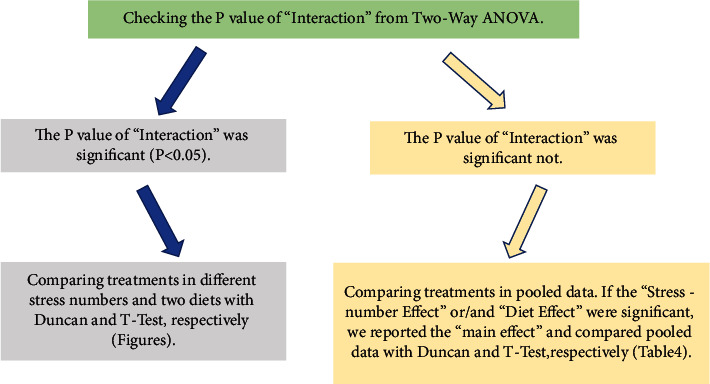
A schematic plot of applied statistical methods in the present study.

This study used a completely randomised design with six treatments and three replications. To investigate the “diet effect” and “stress-number effect,” we used a two-way ANOVA (Figure 1). After ensuring that the data was normal and the variance was homogeneous, the data was analysed. Furthermore, we used an independent sample *T*-test to compare the data before and after stress to see how confinement stress affected each parameter across treatments. The level of 5% was considered the threshold for a significant difference between treatments in all analyses. For data analysis, the SPSS software (version 21.0 for Windows) was used.

## 3. Results and Discussion

The current study is the first aquaculture-based study investigating the positive effects of early mild adaptive stress during a feeding trial to reduce the stress responsiveness of fish after acute stress at the end of the experiment. Interestingly, fish exposed to stressors for two weeks (week 2 and 8) out of ten had a lower degree of stress responsiveness (lower cortisol) after AC stress. When compared to 0Stress treatments, this group displayed equivalent performance. Fish fed high-lipid diets (180 g/kg) outperformed those fed low-lipid diets (100 g/kg), indicating that juvenile oscar requires a high dietary lipid content. There has been no research into the effect of scheduled early stress on measured parameters to compare with current findings. As a result, the data were interpreted with stress-related studies on other fish species.

### 3.1. Growth Performance

One of the most important phenotypes in farmed animals is weight gain. The result of this study represents that “diet effect” was significant and the fish fed high-lipid (HL) diets had higher weight gain (46.41 g) compared to fish fed low-lipid (LL) diets (38.81 g) (*P* < 0.05) (Table 4). Similarly, FCR in the HL and LL groups were 2.21 vs .2.60, respectively. These findings are important because commercially available oscar diets are typically rich in protein and contain less lipid (500 g/kg protein and 100 g/kg lipid) than our diets (400 g/kg protein and 180 g/kg lipid). The protein-sparing effect of lipid has been well documented in carnivorous animals [30–32], and this study is the first time in oscar. Catabolism of proteins produces ammonia, and fish fed with higher protein levels in diet excrete more ammonia which is toxic at excessive levels [33]. Therefore, in any aquaculture system, reduced dietary protein levels would be beneficial for water quality, particularly for ornamental fish reared in aquariums. The number of mild stress events did not affect growth performance; the “stress-number effect” was insignificant for either WG or FCR. When the diet lipid composition was not optimal (LL groups), fish that were subjected to 4Stresses had a lower WG than those not subjected to stress (34.7 g vs. 41.93 g) which may indicate the buffering effect of lipid metabolism. Different carnivorous species have various preferences/abilities to utilise lipids. For example, the optimum dietary lipid content in blunt snout bream (*Megalobrama amblycephala*) [34], tropical gar (*Atractosteus tropicus*) [35], and orange-spotted grouper (*Epinephelus coioides*) [36] was 100 g/kg diet, and that in golden pompano (*Trachinotus ovatus*) was 120 g/kg [37]. However, the optimum dietary lipid levels in salmonids diets were 230 g/kg for rainbow trout [38], 240 g/kg for Coho Salmon (*Oncorhynchus kisutch*) [39], 260 g/kg for triploid brown trout (*Salmo trutta*) [40], 231 g/kg for Chinook salmon (*Oncorhynchus tshawytscha*) (Ridley Corporation) [41, 42], and 280 g/kg for Atlantic salmon [43]. This information shows that oscar is closer to carnivorous freshwater species such as salmonids. The trophic level of oscar is 2.8 compared to rainbow trout, which is 4.1 (https://www.fishbase.de).

Regardless of diet, the survival rate in stressed oscar was higher than that in the 0Stress group. Further, the fish fed HL diets had a higher survival rate (81.25%) than oscars fed the LL diets (72.92%). It is well reported that the optimum lipid levels can increase the survival of fish after stress in shi drum (*Umbrina cirrosa*) [44], Malabar grouper (*Epinephelus malabaricus*) [45], and blunt snout bream [46]. The effect of dietary lipid content on the stress responsiveness of fish was reviewed well [47]. The importance of lipids in stress response is based on the formation of eicosanoids, particularly prostaglandins. Prostaglandins can modulate the stress responsiveness of the hypothalamus-pituitary-adrenal (HPA) axis and eventually cortisol release [47, 48]. However, we could not manage to measure prostaglandins to see how they changed across treatments. To sum up, 180 g/kg lipid optimised the growth rate and survival of fish after AC stress and is recommended for oscar feed formulation.

### 3.2. Hematology and Blood Biochemistry

**Figure 2:**
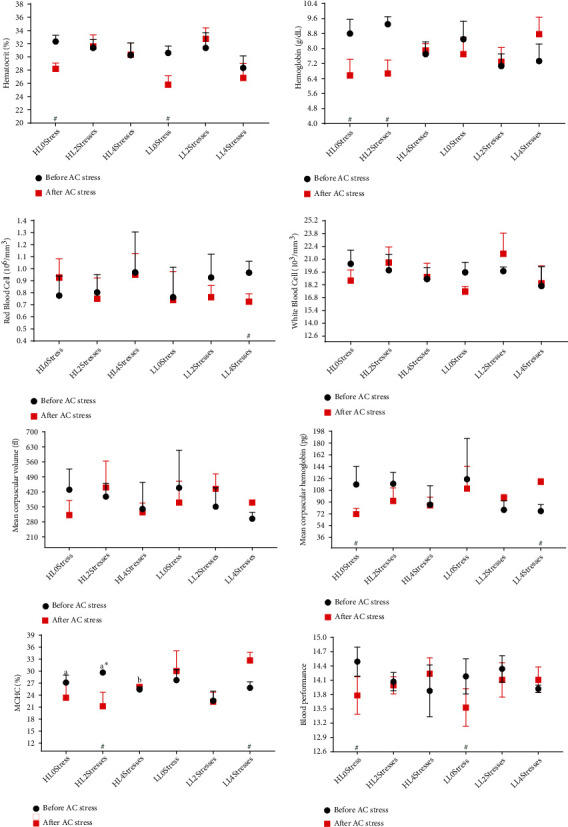
Hematological parameters of oscar fed experimental diets containing different levels of lipid and exposed to scheduled early stress for ten weeks plus data related to after acute confinement stress (AC stress). Values were represented by means ± SDM of triplicate samples. Hash (#) with green colour indicates the significant difference in each treatment before and after AC stress according to the independent sample *T*-test (*P* < 0.05). The six treatments were HL0Stress (high-lipid diet and without stress), HL2Stresses (high-lipid diet and two-week stress), HL4Stresses (high-lipid diet and four-week stress), LL0Stress (low-lipid diet and without stress), LL2Stresses (low-lipid diet and two-week stress), and LL4Stresses (low-lipid diet and four-week stress).

Fish health status has been monitored using hematology and blood biochemistry parameters during environmental and nutritional stressors [49]. According to Table 4 and Figure 2, the diet effect on Hb was significant at the end of the experiment, with individuals fed HL feeds having higher Hb values. These findings can be linked to improved growth performance in these treatments. A higher Hb level may indicate a greater capacity to deliver oxygen to tissues. Previously, a direct relationship between growth performance and Hb content was suggested [50]. Similar to our data, Hb was higher in tiger puffer (*Takifugu rubripes*) fed a diet with optimal lipid levels (the diet that provided maximum growth performance) [51]. Sometimes, hematological parameters do not follow the same trend across treatments, and it is not possible to rely on them as health biomarkers separately. The BP was introduced elsewhere [23] to mitigate this problem, where higher values suggest greater health status. The 4Stresses fish displayed lower BP values, regardless of diet than the other groups, suggesting that the reoccurrence of the stress event surpassed the fish's capacity to cope with it. Beluga sturgeon (*Huso huso*) fed with diets rich in soybean [52] and rainbow trout fed with too much carbohydrate [53] and meat and bone meal [7] exhibited lower BP values than the control group. Furthermore, a lower level of BP was observed when fish were subjected to ammonia stress [7] and heavy metal stress [54]. Many hematological parameters were altered when oscar was subjected to AC stress. The 2Stresses groups had significantly higher Ht, WBC, and BP, as well as lower MCHC compared to 0Stress fish. These findings indicate that the stress responsiveness of 2Stresses fish has been altered in a positive way, as this group better tolerated AC stress by displaying a higher survival rate.

**Figure 3:**
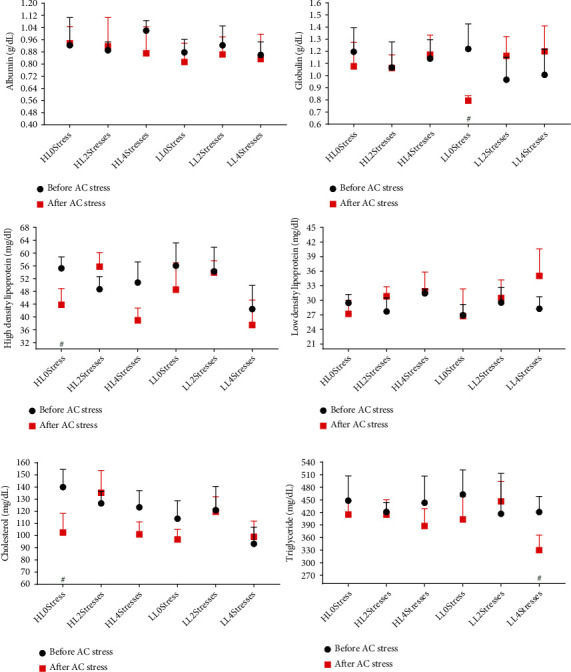
Blood biochemistry parameters of oscar fed experimental diets containing different levels of lipid and exposed to scheduled early stress for ten weeks plus data related to after acute confinement stress (AC stress). Values were represented by means ± SDM of triplicate samples. Hash (#) with green colour indicates the significant difference in each treatment before and after AC stress according to the independent sample *T*-test (*P* < 0.05). The six treatments were HL0Stress (high-lipid diet and without stress), HL2Stresses (high-lipid diet and two-week stress), HL4Stresses (high-lipid diet and four-week stress), LL0Stress (low-lipid diet and without stress), LL2Stresses (low-lipid diet and two-week stress), and LL4Stresses (low lipid diet and four-week stress).

Fish blood biochemistry can reflect the nutritional status as well as the physiological status of the fish. In this study, the cholesterol level was affected by the level of lipids in the diet, with the HL groups presenting higher cholesterol. Cholesterol is involved in many metabolic processes, including cell membranogenesis; growth and cell differentiation; muscle, brain, and nervous system development; bile acids, vitamin D, and many other functions [55]. However, it should be noted that lipid content in diets directly influenced both growth rate and cholesterol content in blood in our study. When fish were subjected to AC stress, the levels of HDL, cholesterol, and triglycerides in 2Stresses treatments were significantly higher than 0Stress and 4Stresses fish (Figure 3 and Table 4). The availability of energy is an important factor in regulating fish stress responses [56, 57]. Lipids and their constituents, such as cholesterol and triglycerides, are the primary energy sources in carnivorous fish species [58]. Higher levels of these parameters indicate that sufficient energy was available for 2Stresses groups, and lower cortisol concentration in these groups supports this hypothesis.

### 3.3. Immune and Stress Response

Diet composition plays a crucial role in the immune system of fish [59, 60]. In the current study, lipid levels did not affect oscar immunity, indicating that the immune system of this fish species is not sensitive to lipid levels as much as other carnivorous fish species. An increase in ACH50, lysozyme, and total protein in blunt snout bream [46] and Malabar grouper after feeding with elevated lipid was observed [45]. More research is needed to show how the immune systems of different fish species react to lipid levels and how this eventually affects growth. Also, testing higher levels of lipid in the oscar diet is suggested.

**Figure 4:**
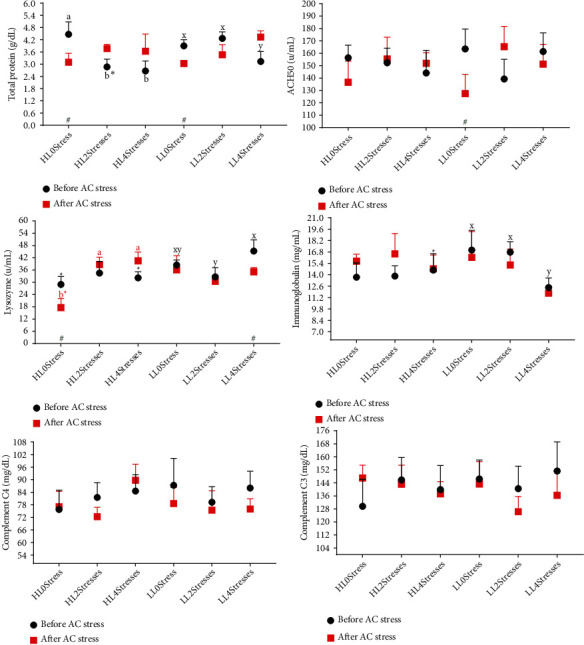
Immune response parameters of oscar fed experimental diets containing different levels of lipid and exposed to secluded early stress for ten weeks plus data related to after acute confinement stress (AC stress). Values were represented by means ± SDM of triplicate samples. Hash (#) with green colour indicates the significant difference in each treatment before and after AC stress according to the independent sample *T*-test (*P* < 0.05). The six treatments were HL0Stress (high-lipid diet and without stress), HL2Stresses (high-lipid diet and two-week stress), HL4Stresses (high-lipid diet and four-week stress), LL0Stress (low-lipid diet and without stress), LL2Stresses (low-lipid diet and two-week stress), and LL4Stresses (low-lipid diet and four-week stress).

Stress has a profound impact on fish homoeostasis, particularly the neural, endocrine, and immune systems. A stimulatory response occurs when the stress is short and acute, whereas a chronic response usually suppresses the immune system [61]. However, the extent to which stress exposure is harmful may vary depending on the severity of the stress, fish species, and the type of stress. In the case of oscar, no research has been conducted to determine how they respond to stress. The current data showed that either the 2Stresses or 4Stresses groups had higher total protein and ACH50 after AC stress than the 0stress treatments (Figure 4 and Table 4). As a result, it is possible to conclude that AC stress suppressed the immune system of oscar. Similarly, AC stress has been shown to suppress the immune system in sea bass (*Dicentrarchus labrax*) [62], rainbow trout [63], Eurasian perch (*Perca fluviatilis*) [64], and Persian sturgeon (*Acipenser persicus*) [65].

**Figure 5:**
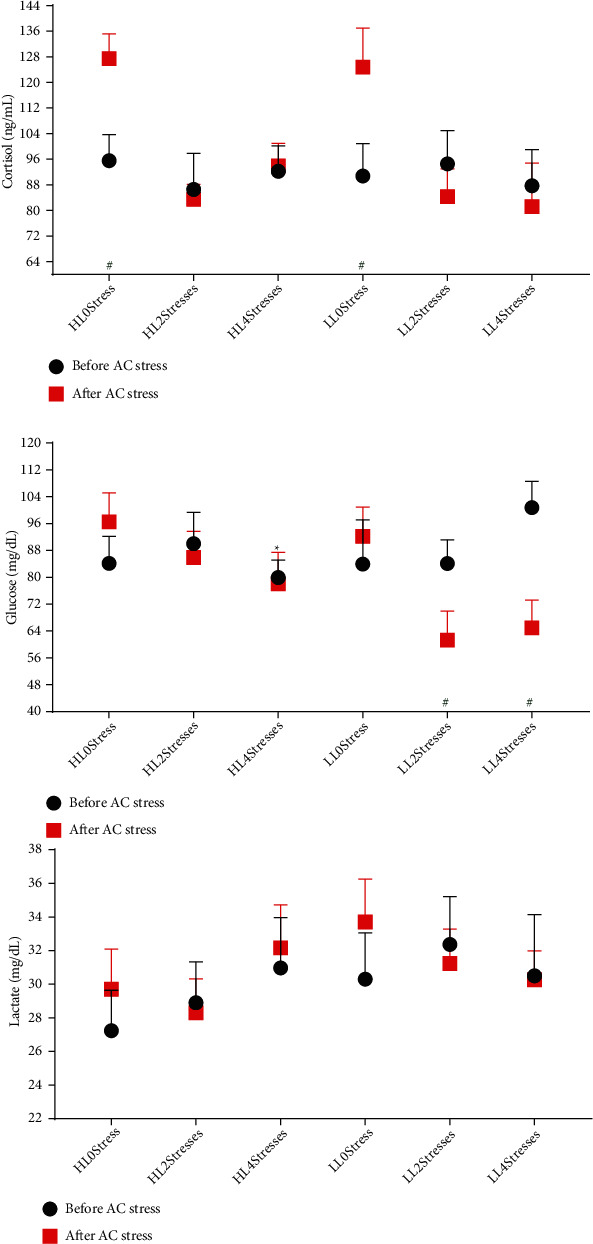
Stress response parameters of oscar fed experimental diets containing different levels of lipid and exposed to secluded early stress for ten weeks plus data related to after acute confinement stress (AC stress). Values were represented by means ± SDM of triplicate samples. Hash (#) with green colour indicates the significant difference in each treatment before and after AC stress according to the independent sample *T*-test (*P* < 0.05). The six treatments were HL0Stress (high-lipid diet and without stress), HL2Stresses (high-lipid diet and two-week stress), HL4Stresses (high-lipid diet and four-week stress), LL0Stress (low-lipid diet and without stress), LL2Stresses (low-lipid diet and two-week stress), and LL4Stresses (low-lipid diet and four-week stress).

Cortisol and glucose are the first and secondary responses of fish to stress, respectively. The concentration of glucose and cortisol can provide valuable complementary information regarding the severity, duration of the stress responses, and the time required for recovery [66]. We hypothesised that early mild stress would help oscar to tolerate the AC stress in later life better. Cortisol responsiveness was one of the most important criteria for testing this hypothesis. Interestingly, after AC stress, glucose, and cortisol levels were lower in stressed groups (2Stresses and 4Stresses) than 0Stress treatments (Figure 5 and Table 4). This decrease in cortisol responsiveness indicates that early stress effectively reduced the negative impacts of AC stress, resulting in a higher survival rate in the 2Stresses groups. Although the 4Stresses group showed good cortisol responsiveness, the survival rate after AC stress in this group was lower than the 2Stresses fish and higher than the 0Stress treatments. Reduced stress responsiveness can improve growth and a higher survival rate during the farming cycle, which is critical for aquaculture sustainability and feasibility. Numerous studies attempted to reduce cortisol responsiveness. One of the most common approaches is supplementing various additives to the fish diet. For example, seaweed in seabass, relaquax in gilthead seabream (*Sparus aurata*) [67], lemon peel (*Citrus limon*) [68], and roselle (*Hibiscus sabdariffa*) [69] mitigated stress and cortisol responsiveness in rainbow trout.

### 3.4. Liver Enzymes

**Figure 6:**
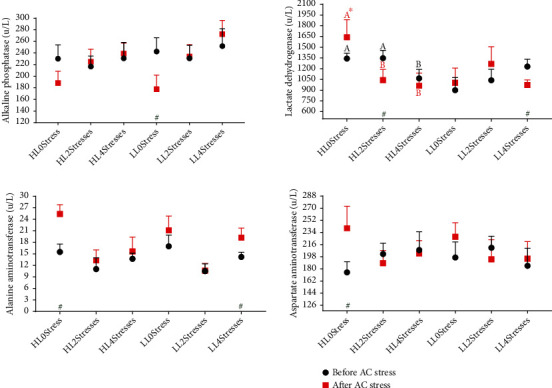
Liver enzyme parameters of oscar fed experimental diets containing different levels of lipid and exposed to secluded early stress for ten weeks plus data related to after acute confinement stress (AC stress). Values were represented by means ± SDM of triplicate samples. Hash (#) with green colour indicates the significant difference in each treatment before and after AC stress according to the independent sample *T*-test (*P* < 0.05). The six treatments were HL0Stress (high-lipid diet and without stress), HL2Stresses (high-lipid diet and two-week stress), HL4Stresses (high-lipid diet and four-week stress), LL0Stress (low-lipid diet and without stress), LL2Stresses (low-lipid diet and two-week stress), and LL4Stresses (low-lipid diet and four-week stress).

The liver is the primary tissue in charge of regulating energy hemostasis in the body, especially during times of stress when more energy is needed [70]. In aquaculture research, liver enzymes or serological enzymes such as LDH, ALP, AST, and ALT are frequently examined to monitor liver physiological status. Further, ALT and AST are involved in the metabolism of amino acids and vitamins, as well as the glucogenesis process [71]. According to Table 4, the dietary composition did not affect liver enzymes, indicating that fish well accepted high-lipid diets. Insufficient or excess dietary lipid levels elevated the ALT and AST values in golden pompano [72]. This showed liver dysfunction in Siberian sturgeon (*Acipenser baerii*) [73] and hybrid snakehead (*Channa argus× Channa maculata*) [74]. The 2Stresses group had lower ALT levels than the other treatments, indicating that this group had slightly greater liver health, which is consistent with improved growth and immunological response. The stress-number effect for ALP, AST, and ALT was significant after AC stress (Table 4 and Figure 6). When compared to stressed treatments, the 0stress group had higher ALT and AST and lower ALP concentrations suggesting hepatic physiological impairments driven by stress. Elevated plasma ALT, AST, and decreased ALP of fish exposed to various types of stress have been well documented [75–79]. According to our findings, fish exposed to early mild stress during the experiment coped well with AC stress, and as a result, liver enzymes were not increased. More research is required to demonstrate the effects of the early stress response on hepatic physiological status.

### 3.5. Antioxidant Enzyme Activities

**Figure 7:**
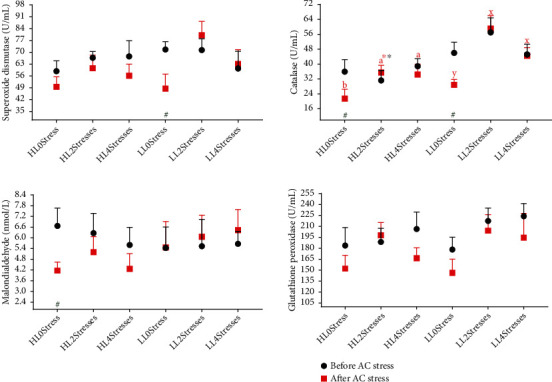
Antioxidant system parameters of oscar fed experimental diets containing different levels of lipid and exposed to secluded early stress for ten weeks plus data related to after acute confinement stress (AC stress). Values were represented by means ± SDM of triplicate samples. Hash (#) with green colour indicates the significant difference in each treatment before and after AC stress according to the independent sample *T*-test (*P* < 0.05). The six treatments were HL0Stress (high-lipid diet and without stress), HL2Stresses (high-lipid diet and two-week stress), HL4Stresses (high-lipid diet and four-week stress), LL0Stress (low-lipid diet and without stress), LL2Stresses (low-lipid diet and two-week stress), LL4Stresses (low-lipid diet and four-week stress).

Studies of antioxidant enzymes such as SOD, CAT, GPx, and MDA compounds in fish can be useful biomarkers for fish physiology under stress or nutritional status. These enzymes play critical roles in protecting cells from uncontrolled oxidative processes that result in superoxide and H_2_O_2_ radical damage [80]. There was no significant difference in antioxidant parameters between treatments in the current study. The only parameter that had a higher value in 4Stresses treatments than in 0Stress treatments was GPx. Most of the changes occurred after AC stress, and individuals fed LL diets had higher SOD and MDA levels than those fed HL feeds. Furthermore, for SOD and GPx, there was a strong stress-number effect, with earlier stressed treatments having higher values than 0Stress groups (Table 4 and Figure 7) (*P* < 0.05). Numerous studies have linked higher antioxidant activities to better health, growth, and physiological conditions, reviewed elsewhere [80]. Higher antioxidant activity in oscar may indicate increased cellular resistance to oxidative stress, the maintenance of the balance between antioxidants and reactive oxygen species (ROS), and, ultimately, a higher survival rate after AC stress. In contrast to our findings, other studies have linked increased antioxidant activity to improved growth performance [50, 81, 82]. Many reasons can drive this inconsistency, such as fish species, size, diet composition, measured parameters.

When fish fed diets with optimum lipid content (in terms of growth rate), antioxidant activities were higher than the control group, for example, in largemouth bass (*Micropterus salmoides*) [83] and *Scylla paramamosain* [84]. However, the same results were not observed in yellow catfish (*Pelteobagrus fulvidraco*) [85], common carp (*Cyprinus carpio*) [86], and Japanese seabass (*Lateolabrax japonicus*) [87], which was in line with our study. The antioxidant system is responsible for removing ROS and avoiding oxidative stress in the body. Steroid components, lipids, and especially unsaturated lipids in cell membranes are more prone to peroxidation [88]. Therefore, providing the optimum level of lipids in fish diet has a direct relation with oxidative stress and the antioxidant system.

## 4. Conclusion

It can be concluded that oscar can accept a diet including 180 g/kg lipid without showing any adverse effects after AC stress. For the first time in aquaculture research, the present findings suggest that scheduled early mild stress can improve fish health and stress responses. The group exposed to two weeks of stress showed better hematology, blood biochemistry, immune responses, antioxidant activities, stress responses, and liver enzyme profiles than control (0Stress), indicating that early life exposure to mild stressors can positively affect fish health. All of these changes were controlled by stress-related mechanisms, indicating that the fish were adapted to stress and had higher survival after being exposed to AC stress. More programmed stresses and analyses at both the classical and molecular levels are required to clarify the various mechanisms underlying early stress responses in fish.

## Figures and Tables

**Table 1:** Formulation and proximate analyses of the experimental diets containing different lipid levels.

Ingredient	High lipid	Low lipid
	g/kg, as-fed basis	
Fish meal	250.0	250.0
Soybean meal	240.0	240.0
Corn meal	161.6	241.6
Meat and bone meal	60.0	70.0
Wheat gluten	120.0	110.0
Fish oil	60.0	30.0
Soybean oil	70.0	20.0
Other ingredients^†^	38.4	38.4
Proximate composition (g/kg dry matter)		
Crude protein	400.5	401.3
Total lipid	179.3	98.4
Ash	70.0	80.5
Carbohydrate^††^	250.0	320.0
Moisture	108.1	105.4
Gross energy (kJ/g)^‡‡^	20.0	19.9

^†^Other ingredients: dicalcium phosphate 3.0 g/kg; mineral premix^†^ 10.0 g/kg; vitamin premix^§^ 10.0 g/kg; antifungus Toxiban premix 10.0 g/kg; antioxidant, butylated hydroxytoluene (BHT) 0.2 g/kg; phytase 0.2 g/kg; lysine 4.0 g/kg; methionine 1.0 g/kg. Diet components were purchased from the Animal & Aquatic Feed store (Arak, Markazi, Iran). ^†^1 kg mineral supplementation contained Co, 100; I, 400; Se, 20; Zn, 10,000; Fe, 6,000; Cu, 600; and Mn, 5,000. ^§^5 kg vitamin supplementation 0.5% contained vitamin A 80,000 IU/kg; vitamin D3 2,000 IU/kg; vitamin k 20 mg/kg; thiamin 60 mg/kg; riboflavin 60 mg/kg; pyridoxine 100 mg/kg; pantothenic acid 150 mg/kg; niacin 300 mg/kg; biothin 2 mg/kg; folic acid 20 mg/kg; vitamin B12 0.1 mg/kg; inositol 300 mg/kg; ascorbic acid 600 mg/kg; and choline chloride 3000 mg/kg. ^††^Carbohydrate = 100 − (crude protein + crude lipid + ash + moisture). ^‡‡^Estimated gross energy was calculated based on 1 g crude protein being 23.6 kj, 1 g crude lipid being 39.5 kj, and 1 g carbohydrate being 17.2 kj. NRC (2011).

**Table 2:** Experimental design for the effect of nutrition and scheduled stress (moving net around the tank after the exchange of water on Monday and Friday of proposed weeks) and acute confinement stress (AC stress) in the end of period.

	HL, without stress	HL, 2-time stress	HL, 4-time stress	LL, without stress	LL, 2-time stress	LL, 4-time stress
Week 1	Without stress	Without stress	Without stress	Without stress	Without stress	Without stress
Week 2	Without stress	Stress	Stress	Without stress	Stress	Stress
Week 3	Without stress	Without stress	Without stress	Without stress	Without stress	Without stress
Week 4	Without stress	Without stress	Stress	Without stress	Without stress	Stress
Week 5	Without stress	Without stress	Without stress	Without stress	Without stress	Without stress
Week 6	Without stress	Without stress	Stress	Without stress	Without stress	Stress
Week 7	Without stress	Without stress	Without stress	Without stress	Without stress	Without stress
Week 8	Without stress	Stress	Stress	Without stress	Stress	Stress
Week 9	Without stress	Without stress	Without stress	Without stress	Without stress	Without stress
	Calculation of weight of all fish, growth, sampling of blood and other factors					
Week 10	Without stress	Without stress	Without stress	Without stress	Without stress	Without stress
Final stress (AC stress) and sampling for serum and hematology parameters.						

**Table 3:** Growth performance of oscar (*Astronotus ocellatus*) fed experimental diets containing different levels of lipid and carbohydrate and exposed to numbers of stress.

	HL0Stress	HL2Stresses	HL4Stresses	LL0Stress	LL2Stresses	LL4Stresses
Initial weight (g)	6.03 ± 0.71	6.90 ± 0.56	6.73 ± 0.72	7.00 ± 0.60	7.03 ± 0.80	7.06 ± 0.95
Weight gain (g)	47.23 ± 8.15	47.00 ± 6.05	45.00 ± 5.23	41.93 ± 3.23	39.80 ± 2.16	34.70 ± 3.04
SGR (%/day)	3.88 ± 0.42	3.66 ± 0.31	3.64 ± 0.27	3.47 ± 0.06	3.39 ± 0.22	3.18 ± 0.14
FCR	2.49 ± 0.23	1.95 ± 0.30	2.21 ± 0.35	2.51 ± 0.34	2.62 ± 0.31	2.66 ± 0.32
DFI (%/day)	7.04 ± 0.32	6.09 ± 0.86	5.37 ± 1.16	6.74 ± 0.98	6.89 ± 0.59	6.75 ± 0.61
HSI (%)	2.65 ± 0.31	2.27 ± 0.25	2.83 ± 0.15	2.20 ± 0.26	2.60 ± 0.20	2.37 ± 0.32
VSI (%)	5.90 ± 0.56	5.33 ± 0.45	5.67 ± 0.55	5.63 ± 0.35	6.13 ± 0.38	5.87 ± 0.61
Condition factor	2.05 ± 0.37	2.16 ± 0.29	2.07 ± 0.22	2.05 ± 0.27	1.84 ± 0.07	1.67 ± 0.28
Survival rate (%)^#^	97.77 ± 3.85	95.55 ± 3.85	93.33 ± 6.67	95.55 ± 3.85	95.55 ± 3.85	95.55 ± 3.85
Survival rate after AC (%)^$^	56.25 ± 8.84	93.75 ± 8.84	81.25 ± 8.84	50.00 ± 0.00	87.50 ± 0.00	68.75 ± 8.84

WG = (final weight − initial weight); SGR: specific growth rate = ((lLn W2 − Ln W1)/63 days) × 100; FCR: feed conversion ratio = dry feed consumed (g)/WG (g); DFI: daily feed intake (%body weight.day^−1^) = 100 × feed consumed (g)/((initial weigh + final weight) × 0.5 × days); HSI: hepatosomatic index = (liver weight (g)/body weight (g)) × 100; VSI : viscerosomatic index = (visceral weight (g)/body weight (g)) × 100; CF: condition factor = (W2 (g)/length3) × 100; ^#^survival rate (%) = (number of fish in each group remaining at the end of experiment/initial number of fish : 20) × 100; ^$^survival rate after 48 hours acute confinement stress (AC stress). The six treatments were HL0Stress (high-lipid diet and without stress), HL2Stress (high-lipid diet and two-week stress), HL4Stress (high-lipid diet and four-week stress), LL0Stress (low-lipid diet and without stress), LL2Stress (low-lipid diet and two-week stress), and LL4Stress (low-lipid diet and four-week stress).Values are represented by means ± SDM of triplicate tanks; means without letter labels are not significantly different.

**Table 4:** The results of two-way ANOVA with SPSS for measured factors. When the interaction was not significant, we compared the “diet effect” via independent samples *T*-test and “stress-number effect” via Duncan's new multiple range test; for the case, these effects were significant (*P* < 0.05). The nonsignificant parameters were not reported. The letters a, b, and c indicate significant differences among treatments exposed to different stress numbers.

	*P* value			Main effects (mean)				
	Diet effect	Stress-number effect	Interactions	HL diet	LL diet	0Stress	2Stresses	4Stresses
WG	0.01	0.28	0.70	46.41	38.81			
FCR	0.02	0.47	0.22	2.21	2.60			
SGR	0.01	0.25	0.82	3.73	3.35			
Survival (%) after AC	<0.01	<0.01	0.49	81.25	72.92	62.50^a^	90.63^c^	78.13^b^
Hb	0.02	0.06	0.07	8.60	7.63			
BP	0.18	0.01	0.85			64.39^a^	63.71^a^	59.13^b^
Ht-stress	0.06	<0.01	0.07			27.00^b^	32.17^a^	28.58^b^
Hb-stress	0.03	0.02	0.86	7.04	7.92	7.13^b^	6.98^b^	8.33^a^
WBC-stress	0.67	0.02	0.49			18.07^b^	21.12^a^	18.71^b^
MCH-stress	0.01	0.51	0.21	82.66	109.03			
MCHC-stress	0.01	<0.01	0.26	23.54	28.35	26.68^a^	21.79^b^	29.36^a^
BP-stress	0.25	<0.01	0.11			56.78^c^	64.64^a^	60.45^b^
TP-stress	0.62	0.02	0.19			3.06^b^	3.82^a^	3.98^a^
Cholesterol	0.01	0.10	0.33	129.99	109.44			
Globulin stress	0.49	0.04	0.12			0.93^b^	1.11^ab^	1.19^a^
HDL-stress	0.86	<0.01	0.56			46.18^b^	54.82^a^	38.20^c^
Cholesterol-stress	0.25	<0.01	0.68			99.75^b^	127.53^a^	100.07^b^
Triglyceride-stress	0.53	0.03	0.22			407.10^b^	430.50^a^	358.60^b^
ACH50-stress	0.99	0.02	0.58			132.02^b^	160.42^a^	151.57^a^
Glucose-stress	<0.01	<0.01	0.17	86.83	72.78	94.35^a^	73.58^b^	71.50^b^
Cortisol-stress	0.29	<0.01	0.46			126.10^a^	83.87^b^	87.55^b^
GPx	0.17	0.03	0.35			181.02^b^	203.03^ab^	215.16^a^
SOD-stress	0.04	<0.01	0.10	55.44	63.89	49.03^c^	70.37^a^	59.58^b^
MDA-stress	0.01	0.42	0.56	4.54	5.99			
GPx-stress	0.36	<0.01	0.41			149.27^b^	200.97^a^	180.43^a^
ALT	0.65	<0.01	0.74			16.23^a^	10.77^b^	13.95^a^
ALP-stress	0.32	<0.01	0.25			182.81^b^	229.38^a^	255.82^a^
ALT-stress	0.44	<0.01	0.08			23.27^a^	11.97^c^	17.42^b^
AST-stress	0.68	0.03	0.81			233.87^a^	191.30^b^	199.00^b^

“-stress” shows the parameter after acute confinement stress (AC stress). WG: weight gain; FCR: feed conversion ratio; SGR: specific growth rate; Hb: hemoglobin; BP: blood performance; Ht: hematocrit; WBC: white blood cell; MCH: mean corpuscular hemoglobin; MCHC: mean corpuscular hemoglobin concentration; TP: total protein; HDL: high-density lipoprotein; ACH50: alternative complement activity; GPx: glutathione peroxidase; SOD: superoxide dismutase; MDA: malondialdehyde; ALT: alanine aminotransferase; ALP: alkaline phosphatase; AST: aspartate aminotransferase.

## Data Availability

Data is available on request due to privacy/ethical restrictions (the data that support the findings of this study are available on request from the corresponding author. The data are not publicly available due to privacy or ethical restrictions).
